# Comparison of pedicle screw placement accuracy between two types of imaging support (Artis Zeego versus two-dimensional fluoroscopy): a cross-sectional observational study

**DOI:** 10.1186/s12891-022-05602-4

**Published:** 2022-07-05

**Authors:** Akira Matsuoka, Tomoaki Toyone, Ichiro Okano, Yoshifumi Kudo, Koji Ishikawa, Hiroshi Maruyama, Tomoyuki Ozawa, Toshiyuki Shirahata, Katsunori Inagaki

**Affiliations:** 1 Department of Orthopaedic Surgery, Showa University School of Medicine, 1-5-8 Hatanodai, Shinagawa-ku, Tokyo, 142-8666 Japan; 2 Department of Orthopaedic Surgery, Tokyo Metropolitan Ohtsuka Hospital, 2-8-1 Minami-Ohtsuka, Toshima-ku, Tokyo, 170-8476 Japan; 3 Department of Orthopaedic Surgery, Tokyo Kyosai Hospital, 2-3-8 Nakameguro, Meguro-ku, Tokyo, 153-8934 Japan

**Keywords:** Spine surgery, Pedicle screw system, 2D X-ray fluoroscopy, Artis Zeego, Cone-beam computed tomography

## Abstract

**Background:**

The pedicle screw system is widely used in spine surgery, and it provides rigid fixation and leads to successful subsequent deformity correction and bony fusion. The standard imaging technique for pedicle screw insertion is two-dimensional images obtained from C-arm-type X-ray fluoroscopy. Artis Zeego is an emerging intraoperative imaging technique that can provide conventional two-dimensional fluoroscopic images and rapid three-dimensional fluoroscopic computed tomography reconstruction imaging. The aim of this study is to compare the insertion accuracies of PS placement using Artis Zeego and conventional 2D X-ray fluoroscopy.

**Methods:**

In this study, we retrospectively reviewed the postoperative images of thoracolumbar fusion patients who underwent surgery using pedicle screws between 2013 and 2018. Pedicle screw malplacement was assessed using a four-grade classification by Rao et al. Misplacement rates were compared between pedicle screws assisted with Artis Zeego and two-dimensional fluoroscopy.

**Results:**

A total of 1107 pedicle screws in 153 patients were inserted using Artis Zeego, and 427 pedicle screws in 80 patients were inserted using fluoroscopy. The overall perforation rate was 4.2% (46 perforations of 1106 pedicle screws) in the Artis Zeego group and 7.7% (33 perforations of 427 pedicle screws) in the fluoroscopy group. In the Artis Zeego group, 43 (3.9%) screws were classified as grade 1, and three (0.3%) screws were classified as grade 2. In the fluoroscopy group, 21 (4.9%) screws were classified as grade 1, 10 (2.3%) screws were classified as grade 2, and 2 (0.5%) screws were classified as grade 3. The use of Artis Zeego was associated with a significantly lower screw malplacement rate than was the use of fluoroscopy (*p* < 0.001).

**Conclusions:**

Our results demonstrated that pedicle screw placement with Artis Zeego was associated with a lower malplacement rate than was conventional two-dimensional fluoroscopy. No severe malplacement was observed in the Artis Zeego group. Thus, Artis Zeego could be a good option for improving pedicle screw accuracy.

## Background

Instrumented fusion has become an indispensable procedure for spinal surgery and has been used for various spinal conditions. The mainstream of posterior instrumentation for fusion is the pedicle screw (PS) system, which provides rigid fixation and leads to successful deformity correction and bony fusion subsequently. However, the posterior PS insertion technique is inevitably associated with a certain risk of injury to the surrounding structures, such as the great vessels, spinal cord, and nerve roots. The risk is generally higher in patients with severe deformities. Although it depends on the definition of malplacement and patient population, the rate of PS malplacement reported in older studies was up to 40% for the lumbar spine and 55% for the thoracic spine [1, 2], whereas in a more recent study, the misplacement rate was lower at approximately 10% [3].

**Fig. 1:**
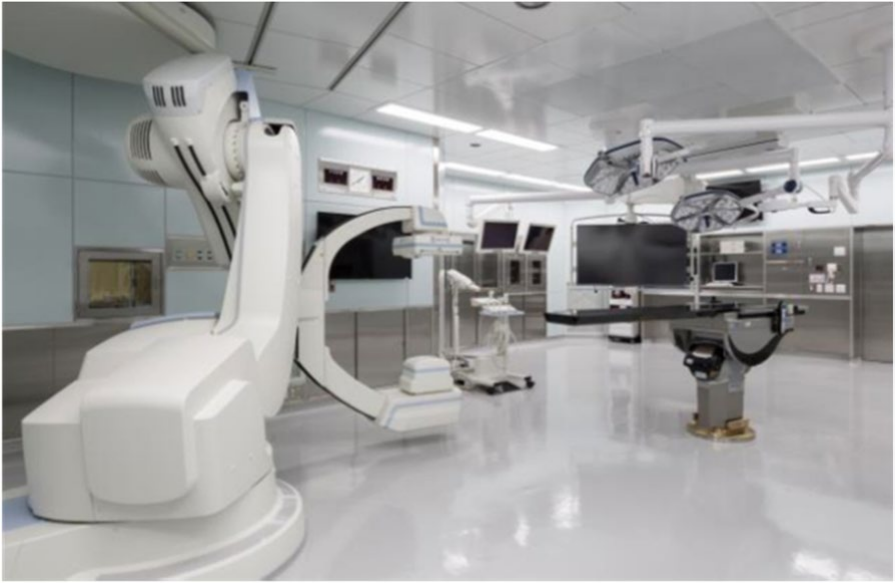
Artis Zeego mounted in the hybrid operating room. (Reprinted with permission from Matsuoka A, Significance of Spine Surgery in the Hybrid Operating Room. Journal of the Showa University Society (Showa Gakushikai Zasshi), 2019 Jun; 79 (3):318–322.)

Previous reports have shown that PS can be safely inserted without imaging support in most patients [4], but recent studies demonstrated that malplacement risk is significantly reduced with appropriate imaging support [5–7]. The standard imaging for PS insertion uses two-dimensional (2D) images obtained from C-arm type X-ray fluoroscopy. Recently, various imaging techniques such as three-dimensional (3D) fluoroscopy or computed tomography (CT)-type have been developed and clinically used with or without a navigation system. Studies have shown that these novel techniques lower the rates of screw malplacement relative to those with conventional 2D fluoroscopy and/or free-hand insertion technique [8–10]. Intraoperative 3D cone-beam CT (CBCT) is an emerging technique, which was first introduced for the surgical planning of craniofacial reconstruction [11], then expanded its indication to major vascular disease, general surgeries, and orthopedic surgery, including the spine [12]. Artis Zeego is a new CBCT originally developed for angiographical treatment [13]. It consists of a floor-mounted multi-axis robotic C-arm and specialized software for image reconstruction (Fig. 1) and is utilized in two ways: conventional 2D fluoroscopy and rapid 3D fluoroscopic CT-like reconstruction imaging. Setting postoperative CT as the gold standard, one previous study demonstrated that intraoperative images using Artis Zeego showed excellent specificity (98%) and acceptable sensitivity (77–79%) for screw malplacement [14].

Although studies have demonstrated that CBCTs, including Artis Zeego, improved the accuracy of PS placement. To the best of our knowledge, no study has directly compared CBCT-based PS placement and conventional 2D fluoroscopy. Therefore, in this study, we compared the insertion accuracies of PS placement using Artis Zeego and conventional 2D X-ray fluoroscopy.

## Materials

### Patient selection

Records and postoperative CT images were reviewed retrospectively from patients who underwent thoracolumbar fusion surgery using percutaneous or open PS, between January 2013 and December 2018 at an academic tertiary care university hospital. Patients with degenerative conditions, trauma, tumor, including metastasis and infections were included, but patients with severe coronal deformity (Cobb angle > 20°) were excluded due to measurement difficulty.

The hybrid operating room (OR) and Artis Zeego were installed in April 2015; thus, we collected the data of consecutive patients who underwent surgery at the hybrid OR between April 2015 to December 2018. We also collected data of patients who underwent fusion surgery with 2D fluoroscopic assistance between January 2013 and December 2015 as the control group.

### Surgical technique of PS insertion

Two-dimensional fluoroscopy or Artis Zeego without a navigation system was used routinely for all instrumentation patients to check the implant positions during the surgery in this study period. All operations were performed by or under direct supervision of a board-certified spine surgeon who had at least five years of spine surgical experience after basic surgical training. The surgical equipment, operative technique, and surgical experience of the operating surgeons were similar during the study period, except for the use of 2D fluoroscopy or Artis Zeego.

For open surgery, the screws were placed using conventional anatomical landmark techniques, and the positions were confirmed using anteroposterior (AP) and lateral images of 2D fluoroscopy or 3D reconstruction images using Artis Zeego. If there were any concerns about screw placement on the intraoperative images, the screws were redirected or removed. For percutaneous PS (PPS) placement with 2D fluoroscopy, cannulated guide needles were placed first using the AP image. After confirmation of the needle positions on the AP view, lateral images were checked, and guide wires were inserted. Cannulated PSs were inserted through the wires using several lateral fluoroscopic images. After the PS insertion, the AP image was checked again before rod placement. For PPS placement with Artis Zeego, the 2D image mode was used and the technique was similar to that with 2D fluoroscopy. Three-dimensional images were checked twice at the end of needle insertion and screw placement, to secure the needle/screw position. If there were any concerns about the needle/screw position, the needles/screws were redirected or removed before connecting the rod. The decision on whether open or percutaneous screw placement was performed was at the surgeon’s discretion.

### Evaluation of screw position of postoperative CT images

Based on the institutional protocol, all patients with instrumentation underwent postoperative CT typically within 1–3 days after surgery. Screw accuracy was assessed using postoperative CT imaging with axial, sagittal, and coronal views. We utilized the grading system of Rao et al. [4], which consists of 4 grades: “0” as no evidence of pedicle perforation, “1” as less than 2 mm of pedicle perforation of the pedicle with one screw thread out of the pedicle, “2” as 2–4 mm of perforation of the pedicle, and “3” as greater than 4 mm (Table 1). While this classification system was originally used for medial and lateral breaches on axial images, we applied the same definition to misplacements on sagittal and coronal planes in any direction. In addition, if the screw was too long, this was defined as “anterior” perforation and graded similarly to the perforation in other directions. If a PS was malpositioned in multiple directions, the highest grade was recorded for each pedicle. For instance, if a PS was misplaced 1 mm laterally (grade 1) and 3 mm caudally (grade 2), we graded the pedicle as “grade 2.”

**Table 1:** Classification of pedicle and anterior vertebral body perforation

	Pedicle perforation	Anterior vertebral body perforation
Grade 0	No violation	No violation
Grade 1	< 2 mm	< 4 mm
Grade 2	2—4 mm	4—6 mm
Grade 3	> 4 mm	> 6 mm

A board-certified spine surgeon who was not directly involved in patient care and was completely blinded for the use of imaging supports, 2D fluoroscopy, and Artis Zeego performed the assessment. In certain situations, the PS was laterally placed intentionally, as per the “in–out-in” technique usually used for small thoracic pedicles. In such pedicles, lateral pedicle wall perforation was not counted. Additionally, we also defined ‘critical perforation,’ (which might cause serious sequalae) as all grade 2 and 3 perforations, except lateral grade 2 and 3 perforations over the thoracic region, as described above.

In the Artis Zeego group, we also investigated the intraoperative revision of the screw, marker, or guide needle, since all intraoperative CT images were stored in the imaging server.

### Statistical analysis

Proportion was used to describe the categorical variables. Mean or median was used to describe continuous variables based on the distribution’s normality. For comparison of categorical variables, the Fisher’s exact or Chi-squared test was used. For comparison of continuous variables, the Student’s t-test or Mann–Whitney U test was used. Statistical analyses were performed using JMP software (version 15.0; SAS Institute, Inc., Cary, NC, USA) or R software (R for 4.0.3, r-project.org).

## Results

### Patient demographics

During the study period, 255 patients underwent spinal fusion under intraoperative imaging using 2D fluoroscopy (80 patients) or Artis Zeego (175 patients). Twenty-two patients were excluded due to a coronal imbalance. A total of 1107 PS in 153 patients were inserted using Artis Zeego, and 427 PS in 80 patients were inserted using 2D fluoroscopy. The mean ages of patients with Artis Zeego and 2D fluoroscopy were similar, 68.5 years and 67.4 years, respectively (*p* = 0.54). Seventy (45.7%) patients in the Artis Zeego group and 32 (40.0%) in the 2D fluoroscopy group were men (*p* = 0.48). Of the 153 patients with Artis Zeego, the diagnoses indicating surgery in the Artis Zeego and 2D fluoroscopy groups were degenerative spine disease in 101 (66.0%) and 48 (60.0%) patients, spinal trauma in 30 (19.6%) and 15 (18.8%), tumor in 11 (7.2%) and 11 (13.8%) patients, and infection in 11(7.2%) and 6 (7.5%) patients, respectively (*p* = 0.44) (Table 2).

**Table 2:** Patient demographics

	Artis Zeego	Two dimensional fluoroscopy	*p*-value
Patient, n	153	80	
Pedicle screw, n	1107	427	
Mean age (years) (range)	68.5(18–88)	67.4(18–84)	0.54
Gender Male (%)	70 (45.8%)	32 (40.0%)	0.49
Number of fused levels			
Median (range)	2.0 (1–12)	2.0 (1–9)	0.11
1 level (%)	51	33	0.50
2 levels (%)	34	15	
3 or more levels (%)	68	32	
Diagnosis			
Degenerative(%)	101(66.0%)	48(60.0%)	0.44
Trauma(%)	30(19.6%)	15(18.8%)	
Tumor(%)	11(7.2%)	11(13.8%)	
Infection(%)	11(7.2%)	6(7.5%)	

### Assessment of screw position

The overall perforation rate was 4.2% (46 perforations of 1106 PS) in the Artis Zeego group and 7.7% (33 perforations of 427 PS) in the 2D fluoroscopy group. In the Artis Zeego group, 43 (3.9%) screws were classified as grade 1, and three (0.3%) screws were classified as grade 2. In the 2D fluoroscopy group, 21 (4.9%) screws were classified as grade 1, 10 (2.3%) screws were classified as grade 2, and 2 (0.5%) screws were classified as grade 3. Regarding the accuracy of PS placement, the use of Artis Zeego was associated with a significantly lower screw malplacement rate than was the use of 2D fluoroscopy (*p* < 0.001). Artis Zeego was also associated with significantly fewer critical perforation (Grade 2 and 3) screws (3/1107 = 0.3% vs. 12/427 = 2.8%, *p* < 0.001) (Table 3). At the lumbosacral level, misplacement most commonly occurred at the S1 pedicle (10/821 = 2.2%) in the Artis Zeego group, whereas the misplacement rate was the highest at L1 (6/30 = 20.0%) in the 2D fluoroscopy group (Table 4).

**Table 3:** Total number of pedicle screws, screws with perforations, and rate of each grade

	Artis Zeego	Two-dimensional fluoroscopy	*p*-values
Pedicle screw, n	1107	427	
Grade 0(rate, %)	1061 (95.8%)	394 (92.3%)	< 0.001
Grade 1(rate, %)	43 (3.9%)	21 (4.9%)	
Grade 2(rate, %)	3 (0.3%)	10 (2.3%)	
Grade 3(rate, %)	0 (0.0%)	2 (0.5%)	
Grade 2–3(rate, %)	3 (0.3%)	12 (2.8%)	< 0.001

**Table 4:** Misplacement rate in both groups stratified by surgical level

	Artis Zeego			2D fluoroscopy		
Level	Number of PS	Re-directed (%)	Misplaced (%)	Number of PS	Misplaced (%)	*p*-values
T1	12	0 (0.0)	1 (8.3)	4	0 (0.0)	1.000
T2	14	0 (0.0)	2 (14.3)	4	0 (0.0)	1.000
T3	8	1 (12.5)	0 (0.0)	2	0 (0.0)	1.000
T4	12	1 (8.3)	1 (8.3)	4	1 (25.0)	0.450
T5	14	1 (7.1)	1 (7.1)	2	1 (50.0)	0.242
T6	15	1 (6.7)	1 (6.7)	8	2 (25.0)	0.269
T7	14	0 (0.0)	2 (14.3)	7	0 (0.0)	0.533
T8	21	3 (14.3)	2 (9.5)	0	-	-
T9	47	4 (8.5)	1 (2.1)	4	1 (25.0)	0.152
T10	68	3 (4.4)	4 (5.9)	14	1 (7.1)	0.534
T11	79	5 (1.3)	0 (0.0)	23	2 (8.7)	0.654
T12	73	7 (9.6)	4 (5.5)	28	1 (3.6)	1.000
L1	71	6 (8.5)	1 (1.4)	30	6 (20.0)	**0.003**
L2	90	6 (6.7)	2 (2.2)	28	2 (7.1)	0.238
L3	119	7 (5.9)	2 (1.7)	46	1 (2.2)	1.000
L4	188	12 (6.4)	5 (2.7)	86	5 (5.8)	0.295
L5	180	18 (10.0)	7 (3.9)	95	5 (5.3)	0.252
S1	82	4 (4.9)	10 (12.2)	42	5 (11.9)	1.000
Total	1107	79 (7.1)	46 (4.2)	427	33 (7.7)	**0.006**

*2D* Two-dimensional, *PS* Pedicle screws

Two-thirds of the pedicle perforations occurred on the axial plane, medially or laterally. There were no patients with cranial malposition in either group (Table 5). Both screws with grade 3 perforation were in the 2D fluoroscopy group, in the S1 pedicles, and perforated medially. These mispositioned screws were asymptomatic, and no patient had neurological deficiency, great vessel injuries, or revision surgery directly related to them.

**Table 5:** Direction and grade of screw perforations

	Lateral			Medial			Anterior			Caudal			Cranial			
Grade	G1	G2	G3	G1	G2	G3	G1	G2	G3	G1	G2	G3	G1	G2	G3	Total
Artis Zeego	14	2	0	13	7	0	0	1	0	9	0	0	0	0	0	46
Two dimensional fluoroscopy	10	4	0	6	3	0	0	3	2	2	3	0	0	0	0	33
Total	24	6	0	19	10	0	0	4	2	11	3	0	0	0	0	79

**Fig. 2:**
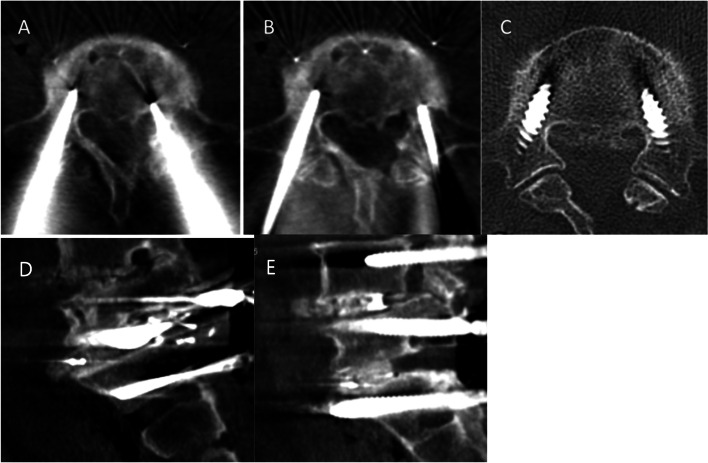
Examples of intraoperative computerized tomography (CT) images. **A** Initial intraoperative image demonstrating a medial misplacement of guiding needle in the left L5 pedicle. **B** Second intraoperative scan image showing the redirected needle properly located in the pedicle. **C** Postoperative CT image showing accurately placed pedicle screws. **D**, **E** Sagittal reconstruction images showing accurately placed needles (**D**) and screws (**E**)

In the Artis Zeego group, a total of 79 PS, markers, or guiding needles (79/1107 = 7.1%) were revised intraoperatively based on intraoperative images. Fifty-two (65.8%), 22 (27.8%), and 5 (6.3%) revised pedicle instruments had medial, lateral, or caudal misplacements on the initial CT images, respectively (Fig. 2).

## Discussion

This study compared the accuracy of PS placement with two imaging supports: Artis Zeego versus conventional 2D fluoroscopy. We found that the screw malplacement rate with Artis Zeego was significantly lower than that with the 2D fluoroscopy guided technique. No grade 3 malplacement was observed in the Artis Zeego group, in contrast to the 2D fluoroscopy group.

Pedicle screw malplacement was reportedly associated with various complications, such as dural tear, nerve and vascular injury, and visceral complications due to the implant itself [15–17]. Moreover, the PS malposition may cause loss of fixation (especially at the lowermost instrumented vertebra), as well as spinal instability [18]. In previous reports, the accuracy of PS placement varied probably due to the definition of malplacement. It has been described as 28–85% with conventional C-arm 2D fluoroscopy, 88–96% with the CT navigation system, and 93–99% with the O-arm navigation system [2, 9, 19, 20]. A meta-analysis of 130 studies with 37,337 PS assessments demonstrated an accuracy of 95.2% with navigation systems and 90.3% without navigation [21]. To the best of our knowledge, only one study has investigated the accuracy of PS placement with Artis Zeego [14]. The main aim of that study was to compare the PS positions between intraoperative images with Artis Zeego and postoperative CT images. Setting postoperative CT images as the reference, the authors reported that the sensitivity, specificity, positive predictive value, and negative predictive value of the Artis Zeego images were 77%, 98%, 86%, and 96%, respectively. They concluded that the Artis Zeego provided an accurate assessment of PS placement, but there was no direct comparison with other imaging supports in their report. In our study, the insertion accuracy with Artis Zeego was 95.8%, which was significantly higher than that with C-arm fluoroscopy. The results were comparable to those of other CT-based techniques, as well as the navigation system. Our results indicate the clinical usefulness of Artis Zeego in patients undergoing lumbar spine surgery.

In our study, the intraoperative needle/marker/PS revision rate in the Artis Zeego group was 7.1%, which was also comparable to that of other CT-guided systems. Bydon et al. reported that they revised 8.97% of screws based on intraoperative CT images [10]. This intraoperative redirection is likely the key factor for superior accuracy of PS with Artis Zeego over 2D fluoroscopy. This feature is useful not only for patient safety, but also for educational purposes. Studies have demonstrated that 80 to over 300 PS insertions are required for spine surgery trainees to reach an adequate level of PS placement [22, 23]. Although further studies are required, immediate intraoperative feedback of the PS position might facilitate the learning of surgical skills.

Interestingly, the level with the lowest accuracy of PS was different between the two groups. The PS malposition most commonly occurred at L1 in the 2D fluoroscopy group, followed by the S1 level. In the Artis Zeego group, the misplacement of L1 was lower, but that of S1 was still high. The L1 vertebra usually has the smallest pedicle, and it seems reasonable to consider it to be more liable to pedicle perforation. In contrast, the S1 vertebra has wider but more angulated pedicles than do the lumbar vertebrae [24–26]. This makes S1 susceptible to lateral and anterior perforations, especially when the medial angulation is insufficient. Additionally, S1 commonly has a “trefoil canal” rather than a cylindrical canal with high lumbar vertebral levels. Reports also mentioned that this characteristic of the spinal canal at S1 could lead to more medial perforation [7]. In our results, the redirection rate was not high at S1 in the Artis Zeego group. This suggests that the guide or marker was placed adequately at the time of intraoperative CT, although the PS could be misplaced more commonly at this level. Since screw insertion instruments are usually larger than the guide or marker, the PS trajectory might have been affected by the iliac crest or surrounding soft tissue. Surgeons should be aware of this, and adequate exposure as well as careful planning of PS insertion, are warranted.

Compared to conventional 2D fluoroscopy, Artis Zeego has more advantages other than the PS insertion accuracy. The main body of Artis Zeego is very stable because it is floor-mounted; moreover, the device has a function that enables it to store data regarding the arm position. In addition, it is easier to operate and requires lesser time than does 2D fluoroscopy to reproduce previously obtained arm positions. This is especially beneficial for percutaneous screw insertion into a highly deformed spine, as it provides optimal image quality. However, the floor-mounted design could also be a disadvantage in certain situations. Unlike conventional 2D fluoroscopy, the relative position of the operating table and Artis Zeego is fixed, and Artis Zeego is always located cephalad to the patient. Therefore, the surgeon needs to stand caudal relative to the arm of Artis Zeego when it is used in the real-time 2D fluoroscopy mode, and this position may not be comfortable for surgeons to perform certain procedures. Additionally, surgeons must use a hybrid OR to use Artis Zeego because of its floor-mounted design. On the other hand, conventional 2D fluoroscopy as well as O-arm and 3D fluoroscopy use portable devices that can be utilized in any OR.

Currently, other CT-based systems are available, and good results have been reported. This study, however, was not a direct comparison study between Artis Zeego and other CT-based systems; therefore, we cannot conclude which is better for patient safety. However, the Artis Zeego demonstrated similar results to previously reported outcomes of other CT-based imaging techniques, and it can be a good option for spine surgery if the system is available to meet the demands of other surgical specialties.

Our study had several limitations. First, this was a retrospective analysis with a relatively small number of patients; thus, the confounding for indication might be an issue. Because of the small number of misplaced screws, statistical power was limited, especially for vertebral level-specific analysis. Additionally, we excluded patients with severe coronal deformity because it was often difficult to assess pedicle perforation using postoperative CT images. With regard to the assessment of CT-based PS misplacement, we used the classification by Rao et al. [4], which has been widely used in similar research. However, Rao et al. reported that the reproducibility of their classification was moderate, and that their CT-based assessment tended to overestimate the degree of misplacement when direct visualization of the specimen was set as the gold standard. Furthermore, because of the unavailability of data, we did not include the details of clinical results, such as pain, radiological fusion, or scores of patient-reported outcome measures. In addition, surgical data such as the screw type, diameter, and length were not available for all cases, and we could not evaluate the influence of parameters such as the screw type on screw misplacement. Lastly, cost-effectiveness and radiation exposure were not analyzed. These points should be addressed in future studies.

## Conclusions

In conclusion, our results demonstrated that PS placement with Artis Zeego was associated with a lower malplacement rate than was conventional 2D fluoroscopy. No severe malplacement was observed in the Artis Zeego group. Therefore, Artis Zeego could be a good option for improving the accuracy of PS.

## Data Availability

The data that support the findings of this study are not deposited in a public repository due to the data sharing policy of the Department of Orthopaedic Surgery, Showa University, but they are available from the corresponding author on reasonable request.

## Abbreviations

2D: Two-dimensional
3D: Three-dimensional
AP: Anteroposterior
CBCT: Cone-beam computed tomography
CT: Computed tomography
OR: Operating room
PPS: Percutaneous pedicle screw
PS: Pedicle screw
